# Social associations and cardiovascular mortality in the United States counties, 2016 to 2020

**DOI:** 10.1186/s12872-024-03749-7

**Published:** 2024-02-26

**Authors:** Ashish Kumar, Kinza Iqbal, Mariam Shariff, Monil Majmundar, Ankur Kalra

**Affiliations:** 1 Department of Internal Medicine, Cleveland Clinic Akron General, Akron, OH USA; 2 Department of Internal Medicine, Dow Medical College, Dow University of Health Sciences, Karachi, Pakistan; 3 Department of Surgery, Mayo Clinic, Rochester, Minneapolis, USA; 4 Department of Cardiovascular Medicine, University of Kansas Medical Center, Kansas City, KS USA; 5 Franciscan Health, Lafayette, IN USA

**Keywords:** Social association, Cardiovascular mortality, Social support

## Abstract

**Background:**

The positive aspects of social interaction on health have been described often, with considerably less attention to their negative aspect. This study aimed to assess the impact of social associations on cardiovascular mortality in the United States.

**Methods:**

The Centers for Disease Control and Prevention Wide-Ranging Online Data for Epidemiologic Research (CDC WONDER) data sets from 2016 to 2020 were used to identify death records due to cardiovascular disease in the United States population aged 15 years and older. The social association rate defined as membership associations per 10,000 population, accessed from the 2020 County Health Rankings data was used as a surrogate for social participation. All United States counties were grouped into quartiles based on their social association rate; Q1 being the lowest quartile of social association, and Q4 the highest quartile. Age-adjusted mortality rate (AAMR) was calculated for each quartile. County health factor rankings for the state of Texas were used to adjust the AAMR for baseline comorbidities of county population, using Gaussian distribution linear regression.

**Results:**

Overall, the AAMR was highest in the 4th social association rate quartile (306.73 [95% CI, 305.72-307.74]) and lowest in the 1st social association rate quartile (266.80 [95% CI, 266.41–267.20]). The mortality rates increased in a linear pattern from lowest to highest social association rate quartiles. After adjustment for the county health factor ranks of Texas, higher social association rate remained associated with a significantly higher AAMR (coefficient 15.84 [95% CI, 12.78–18.89]).

**Conclusions:**

Our study reported higher cardiovascular AAMR with higher social associations in the United States, with similar results after adjustment for County Health Rankings in the state of Texas.

**Supplementary Information:**

The online version contains supplementary material available at 10.1186/s12872-024-03749-7.

## Introduction

Cardiovascular diseases have remained a major cause of morbidity and mortality in the United States and worldwide. In 2019, cardiovascular mortality contributed to around 30% of all deaths in the United States [1]. While there is an extensive amount of literature on the prevention and treatment paradigms of traditional cardiovascular risk factors, recently, there has been an increased focus on elucidating the association of social determinants of health (SDoH) with cardiovascular morbidity and mortality [2–4]. SDoH are defined as the conditions in an individual’s environment, including socioeconomic condition (wealth and income, education, employment/occupational status, and other factors), race and ethnicity, social support (including social networks), culture (including language), religion, healthcare access, neighborhood and environment. Current evidence has implicated them as cardiovascular risk factors and, therefore, are associated with cardiovascular outcomes [5, 6].

Social connections, an integral SDoH, have quantitative and qualitative aspects; both components may influence health to different extent [7]. Social integration is a quantitative measure that includes participation in various social relationships, such as contact with family and friends and involvement in groups or clubs. Social support comprises received and perceived support from social relationships and considers the quality of relationships [8]. Most studies report positive effects of social support and social integration on cardiovascular outcomes [9–13]. A recent study of 11,486 Australians concluded that poor social health increased the likelihood of cardiovascular mortality by two-fold (hazard ratio, HR: 2.00, [95% CI, 1.12–3.60]; *p* = 0.02) [12]. Similarly, a prospective cohort study (1992–2006) that enrolled 5,925 people found that higher social engagement was associated with lower cardiovascular mortality (HR: 0.70 [95%CI, 0.53–0.93]; *p* = 0.0004) [13]. A study reported loneliness to be associated with an increased risk of coronary artery disease and stroke, independent of traditional risk factors, possible secondary to psychological pathways (e.g. depression, anxiety, self-esteem) and/or other behaviors, including alcohol consumption and physical activity [14].

As social relationships are multi-dimensional, it is crucial to gauge the impact of each domain of social relationships on cardiovascular outcomes individually as well as collectively. There is a limited but growing body of research assessing both positive and negative aspects of social support, according to which negative social interaction can have detrimental effects on mental health [15]. According to some studies, negative social interactions may have a greater impact on psychological health than positive interactions [16–18]. Other studies show a more potent effect of positive interactions on psychological well-being [19], whereas some studies found equal effects from the two types of interactions [20]. These conflicting findings prompted us to investigate the impact of social associations rate defined as measure of the number of membership associations per 10,000 population on cardiovascular mortality across the United States counties, stratified by demographic characteristics. We hypothesized that residents of counties with higher social associations will have lower age-adjusted cardiovascular mortality than individuals living in counties with lower social associations.

## Methods

### Cardiovascular mortality

The Centers for Disease Control and Prevention’s Wide-Ranging Online Data for Epidemiologic Research (CDC WONDER) was used to acquire deidentified records of the United States population aged 15 years and older from the “Underlying Cause of Death” datasets from 2016 to 2020 [21]. The Underlying Cause of Death data set includes national mortality and population statistics based on death certificates, and demographic data for the United States counties. We used the International Classification of Disease tenth revision (ICD-10) I00-I78 to identify deaths due to cardiovascular disease, with cardiovascular disease as either an underlying or contributing cause of mortality.

### Social association rate

The social association rate is a metric to assess social or community support at the level of the United States counties. We accessed the public-use data of social association rate from the 2020 County Health Rankings database [22]. Social association rate as stated above is a measure of the number of membership associations per 10,000 population. The numerator is the total number of membership associations in a county, while the total population of a county forms the denominator of this measure. The term “membership association” comprises membership in fitness centers, bowling centers, golf clubs, and civic, sports, religious, political, labor, business, or professional organizations. However, this rate does not take into account the social support offered by families, informal networks, or community service organizations [23].

### Statistical analysis

**Fig. 1:**
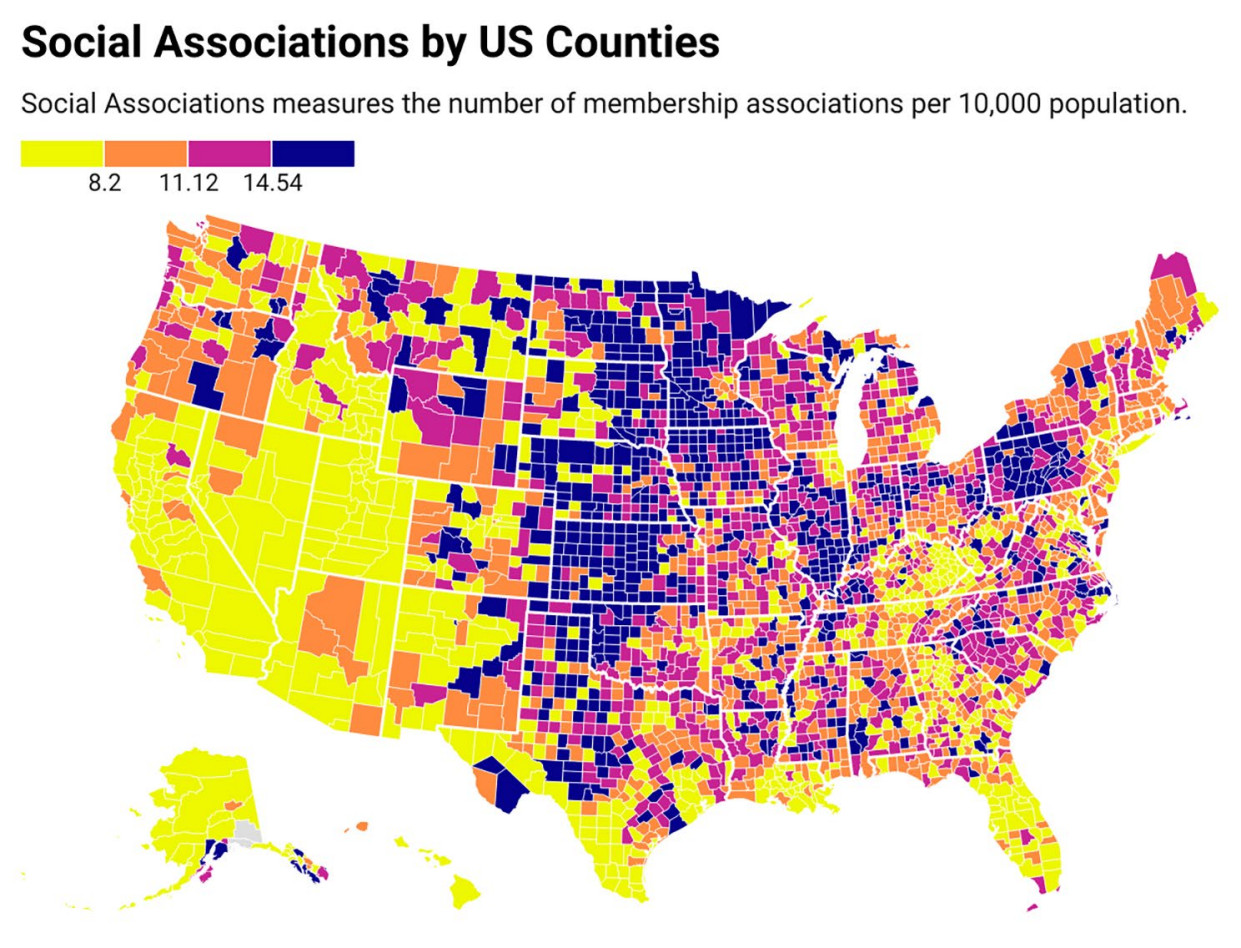
Social associations of all counties in the United States, from the 2020 County Health Rankings database. In the above US heat map, social associations are arranged as quartiles as represented by colors in the figure legend

**Fig. 2:**
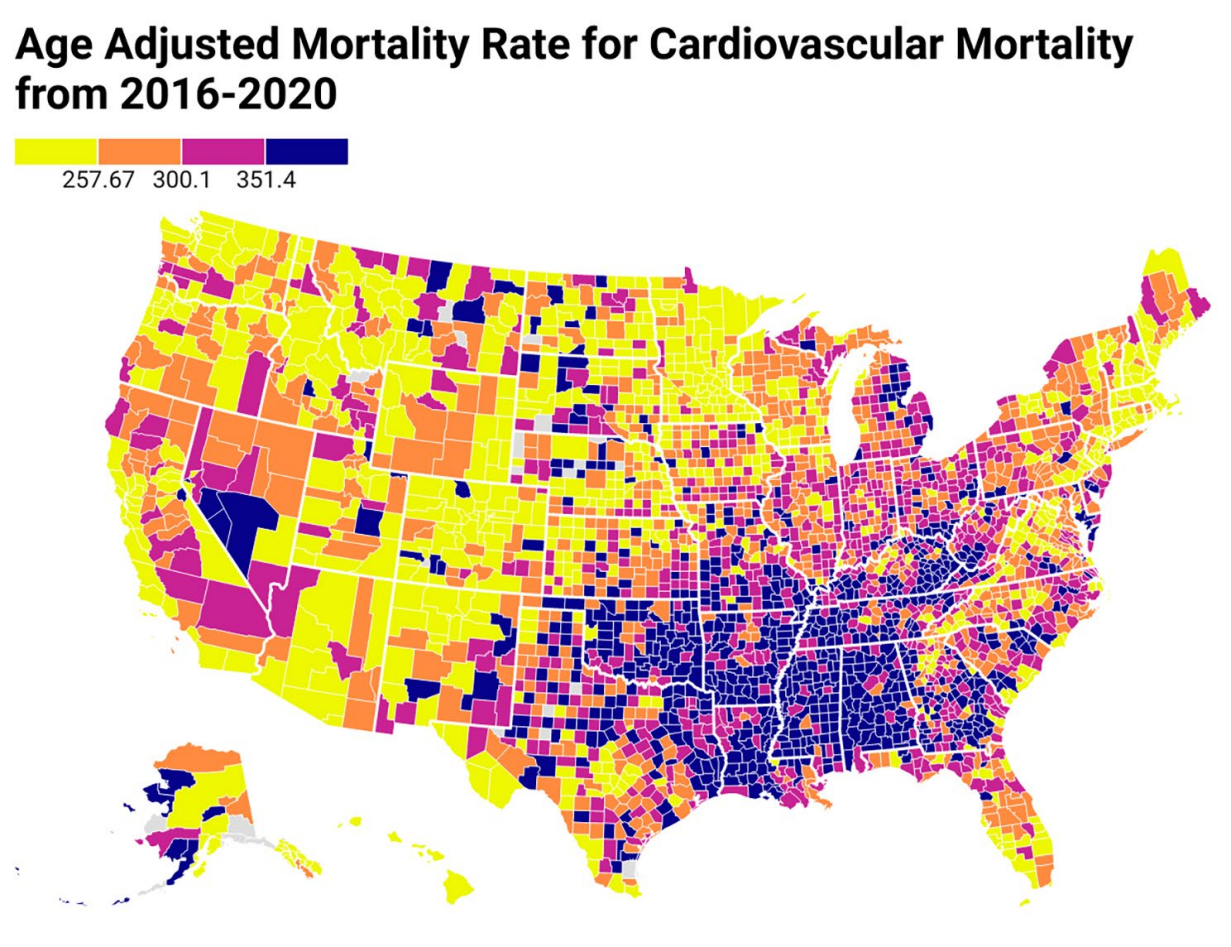
Age-adjusted cardiovascular mortality rates for all counties in the United States from 2016 to 2020. In the above US heat map, age-adjusted cardiovascular mortality rates are arranged as quartiles as represented by colors in the figure legend

All United States counties were grouped into quartiles based on their social association rate; Q1 being the lowest quartile of social association, and Q4 the highest quartile (Fig. 1). Age-adjusted mortality rates per 100,000 population were calculated for each county with a 95% confidence interval using the United States population of the year 2000 as the standard population (Fig. 2). To investigate the association of social association rate with cardiovascular mortality, the age-adjusted cardiovascular mortality rate was calculated for each quartile. Subgroup analyses were conducted based on age (> or < 45), gender (male or female), race (White, Black/African American, Asian and Pacific Islander, American Indian or Alaska Native), ethnicity (Hispanic/Latino or not Hispanic/Latino), urbanization and census region. We grouped the counties into three classes based on 2013 urban-rural classification scheme for counties: large metro (large central metro/large fringe metro), medium-small metro, and non-metro (micropolitan/non-core); the large metro category was the most “urban” category and the micropolitan/non-core category was the most “rural” category [24].

To account for the confounding effect of baseline comorbidities on the association between social association rate and cardiovascular mortality, county health factor ranking was used as a surrogate for baseline comorbidities of county population (Supplementary eMethods) [25]. In short, the state health factor ranking is based on health behaviors, clinical care, social and economic factors and physical environment of counties. Health ranking is available for counties at the state level but not at the national level. Texas was selected as it has the highest number of counties (254 counties), and hence would have the highest degree of freedom in a regression analysis. The county health factor ranking for the state of Texas 2020 was used to adjust the age-adjusted mortality rates (AAMR) for baseline comorbidities of county population using Gaussian distribution linear regression analysis. A *p*-value of < 0.05 was considered to be statistically significant. All analyses were performed utilizing R version 4.0.3 (R Foundation for Statistical Computing, Vienna, Austria). This study was exempt from the institutional review board approval since we used deidentified, government-issued, publicly available datasets. All data extraction from CDC WONDER and statistical analysis were carried out prior to March 7, 2022.

## Results

All 3,143 (100%) US counties were included in the present analysis. The social association of the US counties ranged from 0 to 52.31(Fig. 1). Quartiles of social association rate were scattered throughout the United States, while those with higher CVD mortality were clustered across the southeastern parts of the United States. Between 2016 and 2020, the age-adjusted cardiovascular mortality rate in the United States was 276.82 (95% CI, 276.55-277.08) per 100,000 person-years (Fig. 2). As a comparison, between 2016 and 2020, the age-adjusted all-cause mortality rate in the United States was 938.02 (95% CI, 937.53-938.51) per 100,000 person-years. Overall, age-adjusted cardiovascular mortality rates were higher for adults greater than 45 years of age, men, Black/African American individuals, and non-Hispanic/non-Latino individuals than their counterparts. Non-metro counties had higher age-adjusted cardiovascular mortality rates than metro counties.

### Cardiovascular mortality and social associations

Overall, the age-adjusted cardiovascular mortality rates were highest in the 4th social association rate quartile (306.73 [95% CI, 305.72-307.74]) per 100,000 person-years and lowest in the 1st social association rate quartile (266.80 [95% CI, 266.41–267.20]) per 100,000 person-years. The mortality rates increased in a linear pattern from lowest to highest social association rate quartiles (Table 1). The age-adjusted cardiovascular mortality rates varied considerably across demographic subgroups according to the social association rate quartiles (Table 1).

**Table 1:** Age-adjusted mortality rate per 100,000 for cardiovascular disease, overall, stratified by demographic variables and social associations

	Total	1st Quartile	2nd Quartile	3rd Quartile	4th Quartile
Overall cardiovascular mortality	276.82(276.55-277.08)	266.80(266.41–267.20)	271.44(270.96-271.91)	298.59(297.95-299.23)	306.73(305.72-307.74)
Age in years					
< 45	16.25(16.15–16.35)	14.45(14.31–14.59)	16.35(16.16–16.54)	19.43(19.15–19.71)	21.39(20.90-21.88)
> 45	603.46(602.88-604.04)	583.15(582.29-584.02)	591.21(590.17-592.25)	648.54(647.14-649.95)	664.42(662.23-666.62)
Sex					
Male	335.91(335.46-336.35)	322.95(322.28-323.61)	329.69(328.88-330.49)	364.19(363.09-365.29)	371.77(370.06-373.48)
Female	227.59(227.28-227.91)	219.62(219.15-220.09)	223.52(222.96-224.08)	244.52(243.75-245.28)	251.82(250.62-253.03)
Race					
White	272.51(272.23-272.79)	265.12(264.68-265.55)	266.12(265.61-266.62)	289.36(288.69-290.04)	297.82(296.78-298.86)
Black or African American	360.07(359.10-361.03)	350.57(349.16-351.97)	350.38(348.65-352.11)	386.96(384.62-389.31)	408.94(404.67-413.21)
Asian and Pacific Islander	162.30(161.38-163.21)	169.24(168.14-170.34)	139.89(138.03-141.74)	163.04(164.00-180.48)	149.58(141.14-158.03)
American Indian or Alaska Native	187.86(185.40-190.33)	163.14(159.96-166.32)	205.42(200.33-210.51)	227.01(220.21-233.81)	226.92(215.74–238.10)
Hispanic group					
Hispanic or Latino	204.23(203.46–205.00)	213.17(212.27-214.06)	170.58(168.75-172.42)	179.05(176.09-182.01)	171.20(165.81-176.58)
Not Hispanic or Latino	283.92(283.64–284.20)	276.68(276.24-277.12)	275.91(275.42–276.40)	301.94(301.29–302.60)	309.27(308.24–310.30)
Urbanisation					
Large Metro	263.42(263.07-263.78)	264.68(264.23-265.14)	255.25(254.61-255.89)	278.21(276.93–279.50)	310.58(306.97-314.19)
Medium small metro	279.67(279.19-280.14)	262.81(261.98-263.65)	275.19(274.38–276.00)	297.20(296.28-298.13)	304.76(302.91-306.62)
Micropolitan/NonCore (Nonmetro)	317.03(316.32-317.73)	318.60(316.68-320.51)	324.38(322.96-325.79)	318.16(316.91-319.41)	307.14(305.85-308.42)
Census Region					
Northeast	262.89(262.30–263.48)	282.53(281.45–283.62)	242.55(241.68–243.42)	265.36(263.99–266.72)	288.46(286.17–290.75)
Midwest	288.90(288.32–289.48)	295.20(293.89–296.52)	282.63(281.66–283.60)	292.64(291.56–293.71)	288.74(287.27–290.20)
South	293.62(293.17–294.06)	269.85(269.20–270.49)	297.50(296.67–298.33)	323.41(322.36–324.47)	344.96(343.12–346.80)
West	247.20(246.67–247.72)	250.97(250.37–251.58)	231.62(230.43–232.82)	252.97(250.28–255.65)	222.63(217.77–227.49)

### Age and gender

On subgroup analysis, a similar pattern of linear increase in age-adjusted cardiovascular mortality rate was observed from the lowest to highest social association rate quartiles for men and women (men: 371.77 [95% CI, 370.06-373.48] per 100,000 person-years in the 4th social association rate quartile versus 322.95 [95% CI, 322.28-323.61] per 100,000 person-years in 1st social association rate quartile; women: 251.82 [95% CI, 250.62-253.03] per 100,000 person-years in the 4th social association rate quartile versus 219.62 [95% CI, 219.15-220.09] per 100,000 person-years in the 1st social association rate quartile), and both age groups (age greater than 45: 664.42 [95% CI, 662.23-666.62] per 100,000 person-years in the 4th social association rate quartile versus 583.15 [95% CI, 582.29-584.02] per 100,000 person-years in 1st social association rate quartile; age lesser than 45: 21.39 [95% CI, 20.90-21.88] per 100,000 person-years in the 4th social association rate quartile versus 14.45 [95% CI, 14.31–14.59] per 100,000 person-years in 1st social association rate quartile).

### Race

For most racial groups in the United States, residents of counties with higher social association rate had higher age-adjusted cardiovascular mortality rates (White: 297.82 [95% CI, 296.78-298.86] per 100,000 person-years in the 4th social association rate quartile versus 265.12 [95% CI, 264.68-265.55] per 100,000 person-years in 1st social association rate quartile; Black/African American: 408.94 [95% CI, 404.67-413.21] per 100,000 person-years in the 4th social association rate quartile versus 350.57 [95% CI, 349.16-351.97] per 100,000 person-years in the 1st social association rate quartile; American Indian or Alaska Native: 226.92 [95% CI, 215.74–238.10] per 100,000 person-years in the 4th social association rate quartile versus 163.14 [95% CI, 159.96-166.32] per 100,000 person-years in the 1st social association rate quartile; non-Hispanic/Latino: 309.27 [95% CI, 308.24–310.30] per 100,000 person-years in the 4th social association rate quartile versus 276.68 [95% CI, 276.24-277.12] per 100,000 person-years in the 1st social association rate quartile). On the contrary, for Asian/Pacific Islander and Hispanic individuals, higher social association rate was associated with lower age-adjusted cardiovascular mortality rates (Asian/Pacific Islander: 149.58 [95% CI, 141.14-158.03] per 100,000 person-years in the 4th social association rate quartile versus 169.24 [95% CI, 168.14-170.34] per 100,000 person-years in the 1st social association rate quartile; Hispanic: 171.20 [95% CI, 165.81-176.58] per 100,000 person-years in the 4th social association rate quartile versus 213.17 [95% CI, 212.27-214.06] per 100,000 person-years in the 1st social association rate quartile).

### Urbanicity and census region

People residing in metro counties with higher social association rate had higher age-adjusted cardiovascular mortality rates than the residents of metro counties with lower social association rate (large metro counties: 310.58 [95% CI, 306.97-314.19] per 100,000 person-years in the 4th social association rate quartile versus 264.68 [95% CI, 264.23-265.14] per 100,000 person-years in the 1st social association rate quartile; medium/small metro counties: 304.76 [95% CI, 302.91-306.62] per 100,000 person-years in the 4th social association rate quartile versus 262.81 [95% CI, 261.98-263.65] per 100,000 person-years in the 1st social association rate quartile). However, residents of non-metro counties with higher social association rate had lower age-adjusted cardiovascular mortality rates than those residing in non-metro counties with lower social association rate (307.14 [95% CI, 305.85-308.42] per 100,000 person-years in the 4th social association rate quartile versus 318.60 [95% CI, 316.68-320.51 per 100,000 person-years in 1st social association rate quartile). With regards to the census region, only in the South census region, higher social association was associated with higher age-adjusted cardiovascular mortality; South census region: 344.96 [95% CI, 343.12–346.80] per 100,000 person-years in the 4th social association rate quartile versus 269.85 [95% CI, 269.20–270.49] per 100,000 person-years in the 1st social association rate quartile.

### Adjustment for comorbidities

**Fig. 3:**
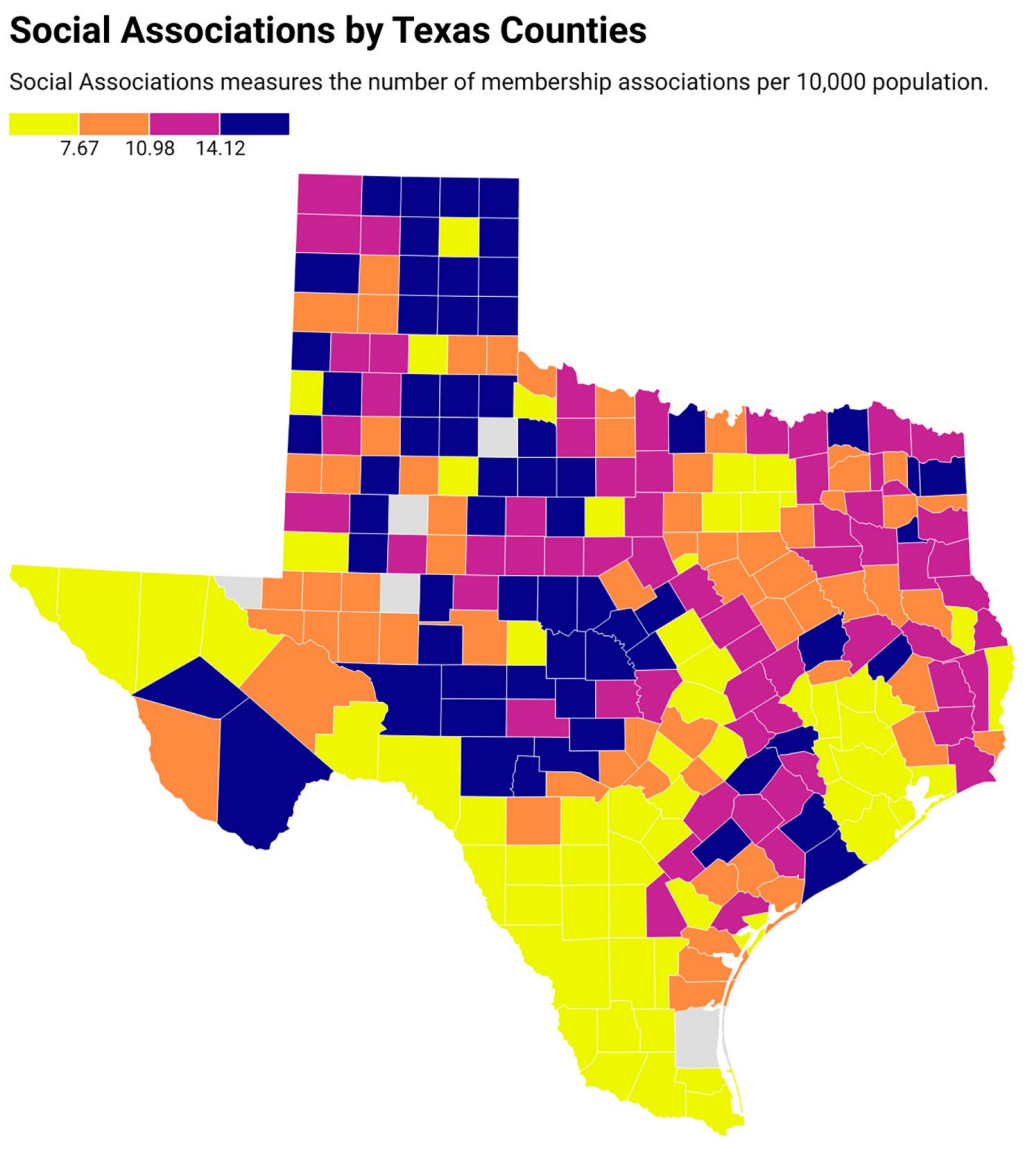
Social associations all counties in Texas from the 2020 County Health Rankings database. In the above Texas heat map, social associations are arranged as quartiles as represented by colors in the figure legend

**Fig. 4:**
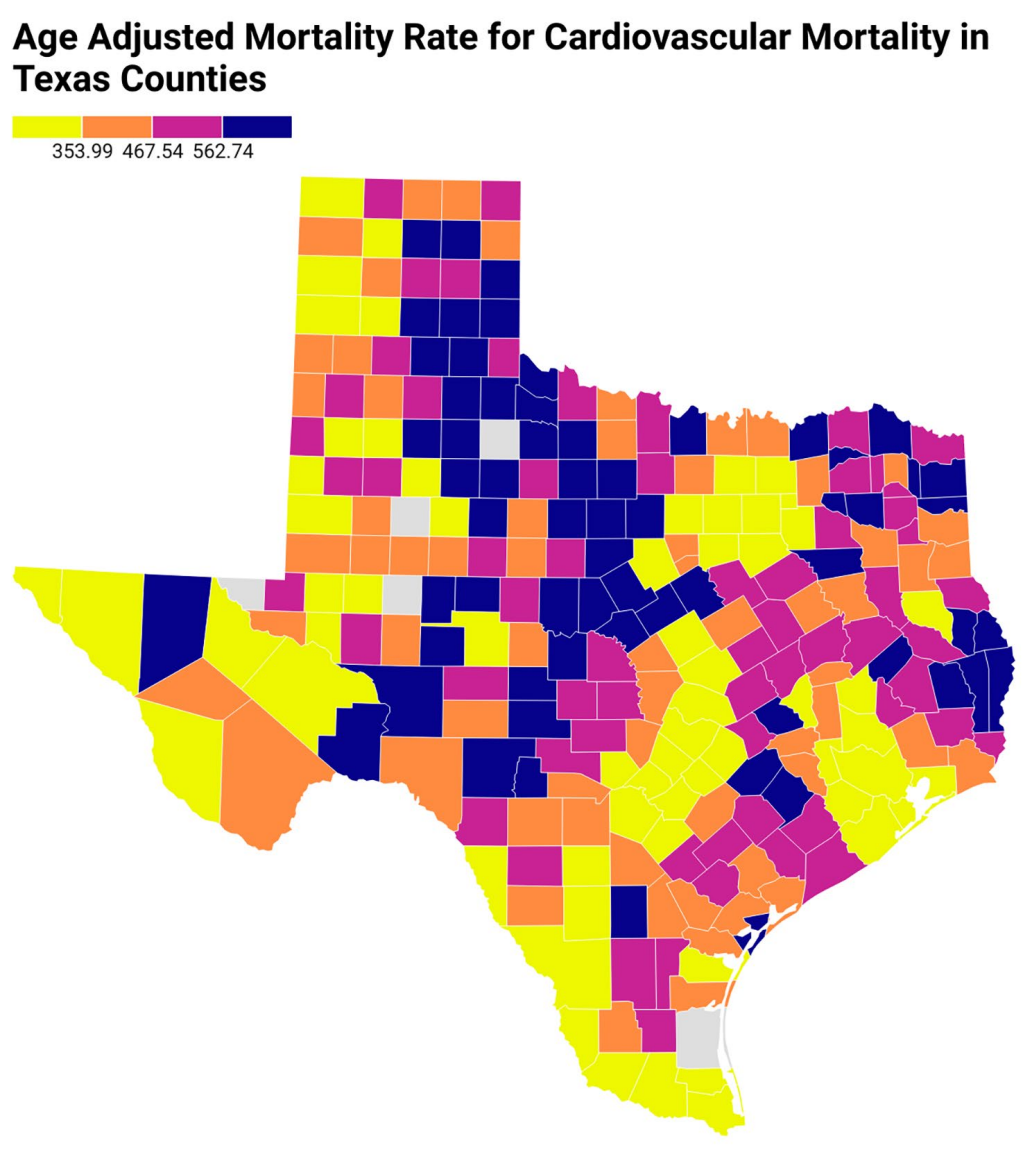
Age-adjusted cardiovascular mortality rates for all counties in Texas from 2016 to 2020. In the above Texas heat map, age-adjusted cardiovascular mortality rates are arranged as quartiles as represented by colors in the figure legend

**Fig. 5:**
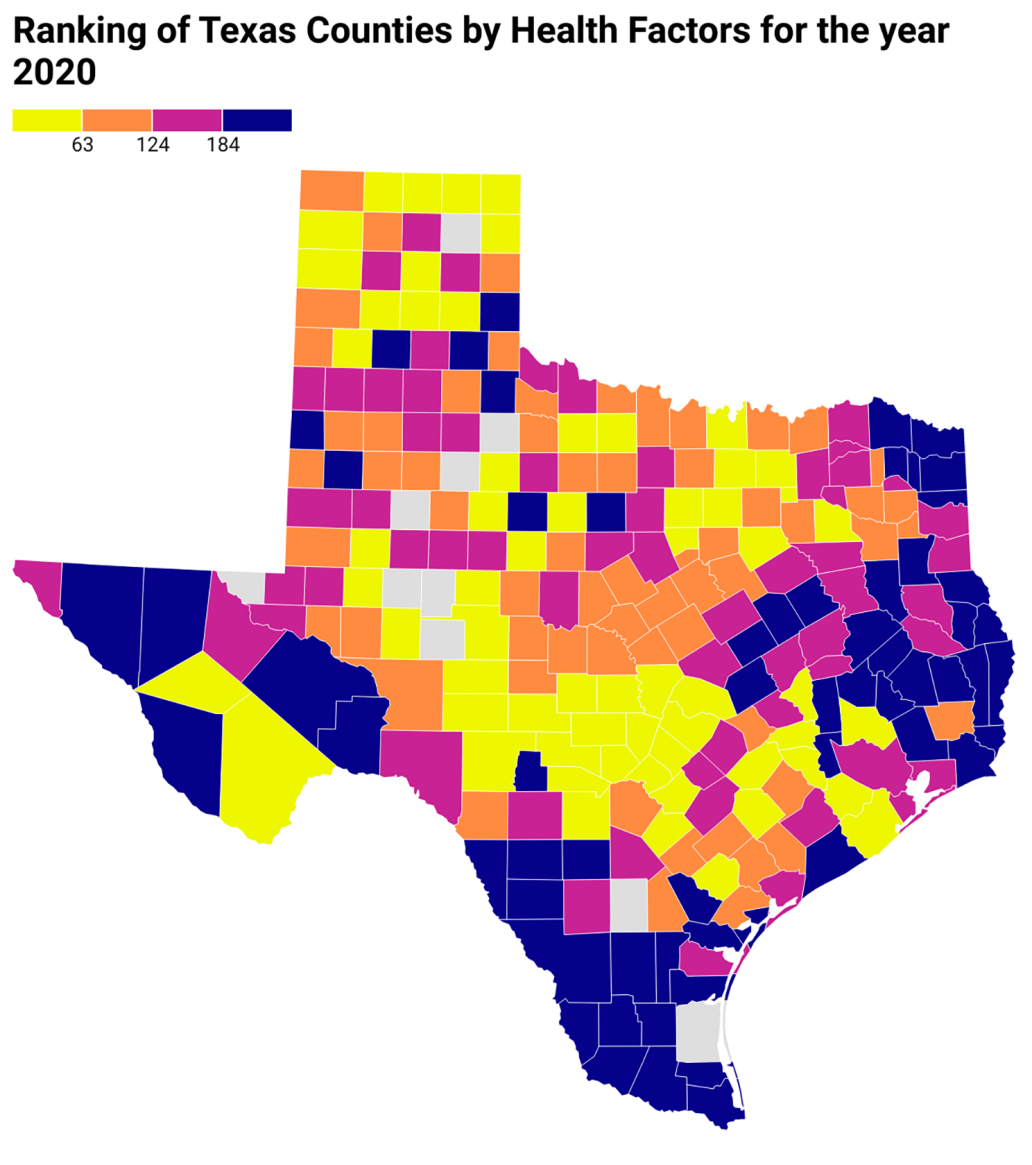
Ranking of the counties in Texas by health factors per 2020 County Health Rankings database. In the above Texas heat map, counties are arranged as quartiles based on health factor rankings as represented by colors in the figure legend

Figure 3 presents the social association rate of the counties in Texas, while Fig. 4 depicts the age-adjusted cardiovascular mortality rates for all Texas counties. After adjustment for the county health factor ranks of Texas (Fig. 5) using linear regression, with county health factor ranks as numeric variables, a higher social association was still associated with significantly higher age-adjusted cardiovascular mortality rate with a coefficient of 15.84 (95% CI, 12.78–18.89).

## Discussion

In this analysis of the United States population from 2016 to 2020, counties with higher social associations had higher age-adjusted cardiovascular mortality rates across both genders, age groups, most racial groups, metro counties, and South census region. However, Asian and Hispanic residents of counties with higher social associations had lower age-adjusted cardiovascular mortality rates for unknown reason. Non-metro counties with higher social associations had lower age-adjusted cardiovascular mortality rates than non-metro counties with lower social associations. Even following adjusting for baseline comorbidities of county population using the data of counties in Texas, higher social associations remained significantly associated with higher age-adjusted cardiovascular mortality rates.

Through the lens of SDoH, health, illness, and resources to prevent illness are not distributed randomly in the society, rather they are driven by socio-economic, healthcare, and environmental forces [1]. SDoH have been shown to influence cardiovascular events and mortality. A recent study assessed the impact of SDoH on 90-day mortality in 690 patients hospitalized for heart failure. It was noted that having one SDoH (HR: 2.89 [95% CI, 1.46–5.72]) or ≥ 2 SDOH (HR: 3.06 [95% CI, 1.51–6.19]) increased the likelihood of 90-day mortality compared with having no SDoH [26]. An analysis of around 303,036 individuals from Asia and Australasia showed that individuals with primary education had an increased risk of cardiovascular mortality compared with those with tertiary education (HR: 2.4 [95% CI, 1.47, 4.17], Australasia: HR: 1.24 [95% CI 1.02, 1.51]) [27]. A United States cohort study reported an increased risk of a cardiovascular event or cardiovascular mortality in individuals who experienced downward wealth mobility compared with those who had stable wealth (HR: 1.15 [95% CI, 1.00-1.32]; *P* = 0.046) [28]. Moreover, a cohort study with 15,000 participants found that uninsured individuals had an increased risk of mortality relative to the ones with health insurance (HR: 1.26, [95% CI 1.03–1.53]) [29]. Similarly, food security, housing stability, neighborhood socioeconomic conditions have also been linked with cardiovascular events and mortality [30, 31].

Social support and social integration are the components of SDoH that have been studied in association with cardiovascular mortality. In contrast to our hypothesis, we observed that having higher social associations significantly increased cardiovascular mortality. This finding can be attributed to the fact that the measure of social associations used by the United States county health rankings did not include contact with family and friends, and was restricted to involvement in membership groups or clubs. Social interactions in membership groups or clubs are not always beneficial for an individual’s well-being. The impact of these associations on a person’s health hinges on the quality of support received in membership clubs. Despite being a member of several organizations and clubs, individuals may still feel unwelcomed, discriminated against, or out of place in such clubs, which can negatively impact their mental health and, subsequently, their physical health. Some members may continue their club memberships to blend with their communities despite the stress and anxiety caused by them. Our study results are in agreement with a study which documented self-reported loneliness rather than social isolation being associated with increased risk of coronary artery disease and stroke at follow-up [14].

A longitudinal study of 2,328 participants from rural Malawi (2008–2010) showed mixed results for the association between social participation and health. Higher overall monthly social engagement in 2008 was linked to improved physical health for both women (*p* < 0.05) and men (*p* < 0.10) in 2010. However, greater annual participation in 2008 was associated with lower Social Functioning (SF-12) mental health scores for women (*p* < 0.05) and men (*p* < 0.10) in 2010. Memberships in a greater number of groups in 2008 were not linked with mental and physical health in 2010 for women or men [32]. A qualitative study enrolling 30 women was conducted in Australia to gain insights into the negative consequences of participation in diverse community groups. Of these 30 women, fourteen reported negative experiences; many women felt overwhelmed by managing their family and work responsibilities alongside social participation. Some women even felt guilty about sparing time for these clubs instead of tending to their families. These pressures can overburden women and such social interactions take a toll on their mental health [33]. Receiving social support may involve obligations of reciprocity and a feeling of indebtedness that can affect the recipient’s mental health [34]. In addition, a cross-sectional analysis of the effects of social participation on the health of 12,132 elderly in Japan noted that obligatory participation decreased mental health component summary scores compared with voluntary participation, and in some cases, compared with non-participation [35]. Similarly, a study of 222 residents of an impoverished community found that social participation can have a deleterious impact on mental health as it can become cumbersome and an additional obligation for an individual with an already stressful daily routine [36]. Psychological distress and anxiety have been linked with cardiovascular mortality [37]. A meta-analysis of 46 cohort studies showed that anxiety was associated with a higher risk of cardiovascular mortality (relative risk RR:1.41, [95% CI 1.13 to 1.76]) [38]. Several mechanisms have been postulated for the association of mental stress with cardiovascular mortality, including increased sympathetic activity, which can increase ambulatory blood pressure and heart rate, reduced insulin sensitivity, increased platelet aggregation, and endothelial dysfunction [39].

Most studies have focused on the positive aspects of social support obscuring the dark side of social relationships. Tense, conflicted, or overly demanding social relationships can contribute to added stress and strain, canceling out the ameliorative effects of social support. At times, even support extended with pure intentions can offend or distress recipients instead of providing comfort [40, 41]. Moreover, one’s social circle, especially in adolescence, can influence unhealthy practices such as smoking and alcohol consumption, thus exerting an indirect influence on cardiovascular outcomes [42]. Routine adverse health behaviors in membership clubs like smoking and consumption of alcohol or junk food can act as precipitants for cardiovascular disease over time. Another possible explanation of the negative influence of social support on cardiovascular mortality could be the impingement of an individual’s sense of personal mastery. By definition, personal mastery is a global sense of control or autonomy in future important life events. Excessive social support can induce dependency; a reduced sense of control and autonomy can dent a person’s psychological as well as physical well-being [43–45]. Evidence shows higher cardiovascular mortality in individuals with lower personal mastery [46, 47]. A review of 32 studies exploring the association of personal mastery with cardiometabolic health outcomes found that 24 studies reported higher personal mastery positively influenced cardiovascular outcomes or health in general [44]. Measures of optimal psychological functioning have been linked with decreased plasma levels of inflammatory biomarkers and with reduced cardiovascular and mortality risk. Different levels of personal mastery manifest as variation in the ability to cope with stressful events and willingness to adopt healthy lifestyle behaviors [47].

Less attention is given to negative impact of social interaction in the existing literature. Instead of idealizing the role of social support on health, a more realistic approach is needed. Social workers should comprehensively assess both the quantity and the quality of social interactions to gauge the impact of social support on health. The mixed results of social participation suggest that it should not be considered a public health strategy or a substitute for medical care. Rather, researchers should continue to investigate why certain types of social interactions improve or worsen different aspects of health, with the aim to identify ways in which social participation can complement the provision of healthcare services. Our study calls for the integration of negative aspects of social relations in social support assessment instruments. Future research should explore how social interactions may vary across age groups, races, and socioeconomic conditions. Further research is needed to elucidate the nuanced pathways linking negative social relations with increased cardiovascular mortality.

The strengths of our study include its novelty in assessing the impact of membership associations with cardiovascular mortality on a national level using the CDC WONDER Database. Moreover, mortality rates were adjusted for age and baseline comorbidities and residual confounding attributable to these variables was accounted for. Nevertheless, there are some limitations in the present analysis that should be noted. As we did an analysis controlling for baseline comorbidities using county health rankings of Texas, we acknowledge that counties outside of Texas were excluded for this analysis, which could potentially attenuate our findings. Potential confounding effects of other SDoH on cardiovascular mortality were not accounted for. In addition, social support is a broad concept; the social association rate used in this analysis does take into consideration the important social connections offered via family support, informal networks, or community service organizations. The social association rate also does not take perceived support into account. Being a part of many social associations does not guarantee social support as some members may feel unwelcomed or discriminated against in these clubs. We did not account for social support derived from social media or its possible deleterious effects on cardiovascular outcomes. Racial and ethnic segregation of membership associations were not accounted for in the current manuscript. Factors like political views and religious participation were not accounted for in the present analysis. Due to the cross-sectional design of this analysis, it was not possible to establish causality. Individual-level inferences cannot be made as this was an aggregate-level analysis at the level of counties rather than for each individual having a death record in the CDC WONDER database. The possibility of misclassification of the cause of death in this database cannot be ruled out.

## Conclusion

The positive impact of social relationships on health has been described so often that the detrimental effects of some social interactions are frequently brushed over. Reports of negative social interactions, although rare, are of significance as they highlight the dire need to consider both the positive and negative effects of social relationships in tandem. Comprehensive assessment of both the quantity and quality of social interactions is necessary to evaluate the impact of social relationships on health. It is imperative to include negative aspects of social relations in social support assessment instruments. Further research into the varied role of social associations across different races and socioeconomic groups is warranted.

### Electronic supplementary material

Below is the link to the electronic supplementary material.

Supplementary Material 1

## Data Availability

The datasets used and/or analysed during the current study are available from the corresponding author on reasonable request.

## Abbreviations

SDoH: social determinants of health
AAMR: age-adjusted mortality
HR: hazard ratio
RR: risk ratio
