# Supplementary material for: Social associations and cardiovascular mortality in the United States counties, 2016 to 2020
Source: BMC Cardiovasc Disord. 2024 Feb 26;24:127. doi: 10.1186/s12872-024-03749-7 (PMC10898153; doi:10.1186/s12872-024-03749-7)

**SUPPLEMENTARY FILE**

**eMethods:**

We used county level health factors for the state of Texas to adjust the age-adjusted mortality rates (AAMR) for baseline comorbidities of county population. The county level health factor ranking along with county level health outcome ranking is used to report the county health ranking, based on a model of community health that emphasizes factors that influence the quality and quantity of our life. Derivatives of the county health outcome ranking include the length of life (50%) and quality of life (50%), while the derivatives of the county health factor ranking include, health behaviors (30%), clinical care (20%), social and economic factors (40%) and physical environment (10%). Numbers in brackets represent weights of the factors in ranking and was determined by literature review, ability for health factors to be modified through community action, review of America's Health Rankings methodology and indicators, availability and reliability of nation-wide, county-level data, internal analysis, and a panel of technical experts and scientific advisor. Further, counties and county equivalents are only ranked within their respective states. Since health varies between places, there could be concerns with data comparability and availability across states and hence counties are only ranked within states.

**Figure s1: Health behaviors ranking of counties in Texas in the year 2020, counites grouped in quartiles with darker shades representing higher quartiles( higher in health behaviors ranking)**


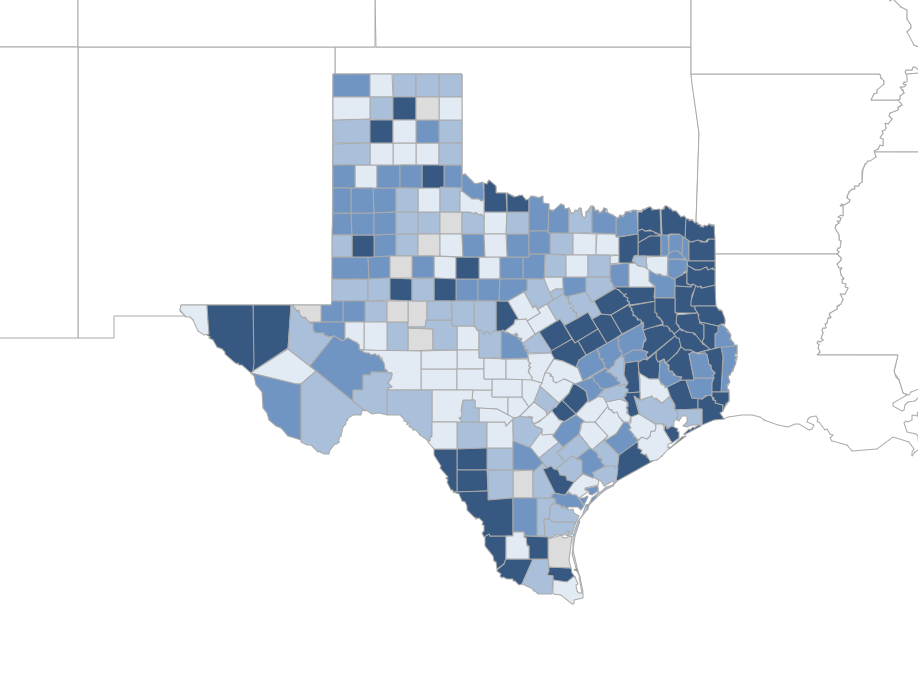


**Figure s2: Clinical care ranking of counties in Texas in the year 2020, counites grouped in quartiles with darker shades representing higher quartiles( higher in clinical care ranking)**


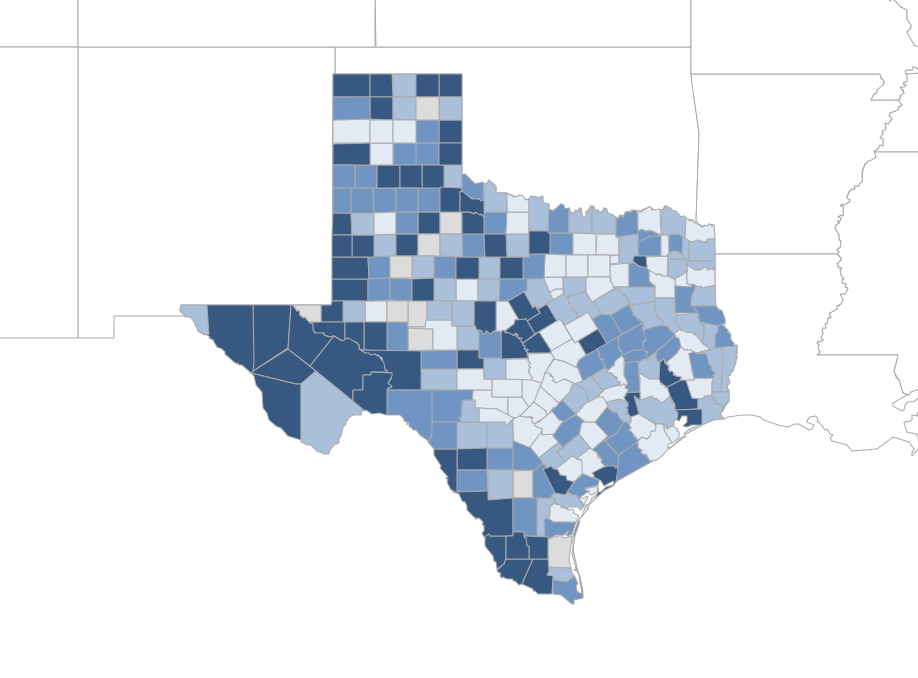


**Figure s3: Social and economic factors ranking of counties in Texas in the year 2020, counites grouped in quartiles with darker shades representing higher quartiles( higher in social and economic factors ranking)**


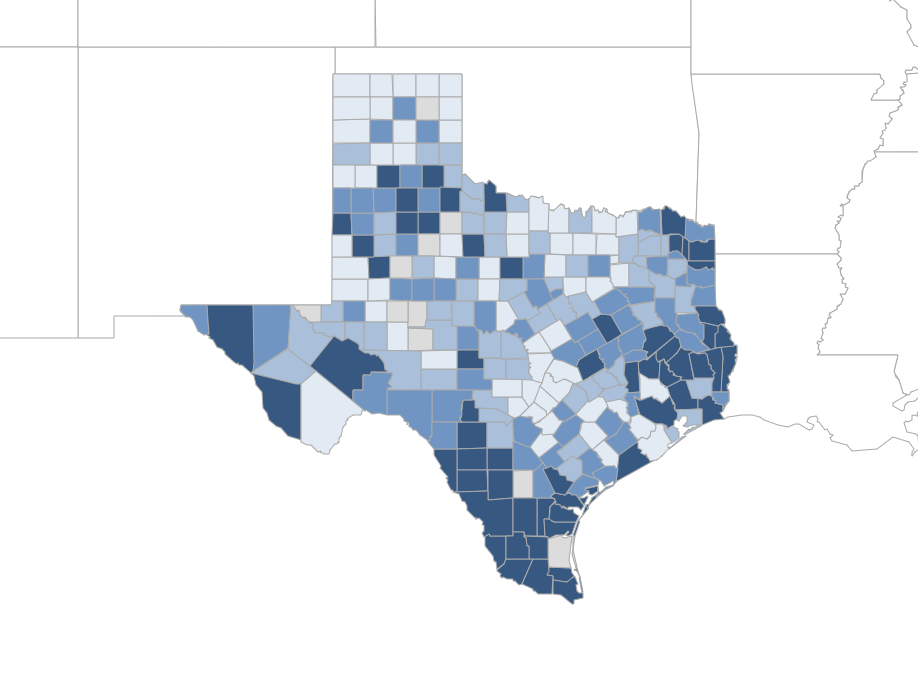


**Figure s4: Physical environment ranking of counties in Texas in the year 2020, counites grouped in quartiles with darker shades representing higher quartiles( higher in physical environment ranking)**


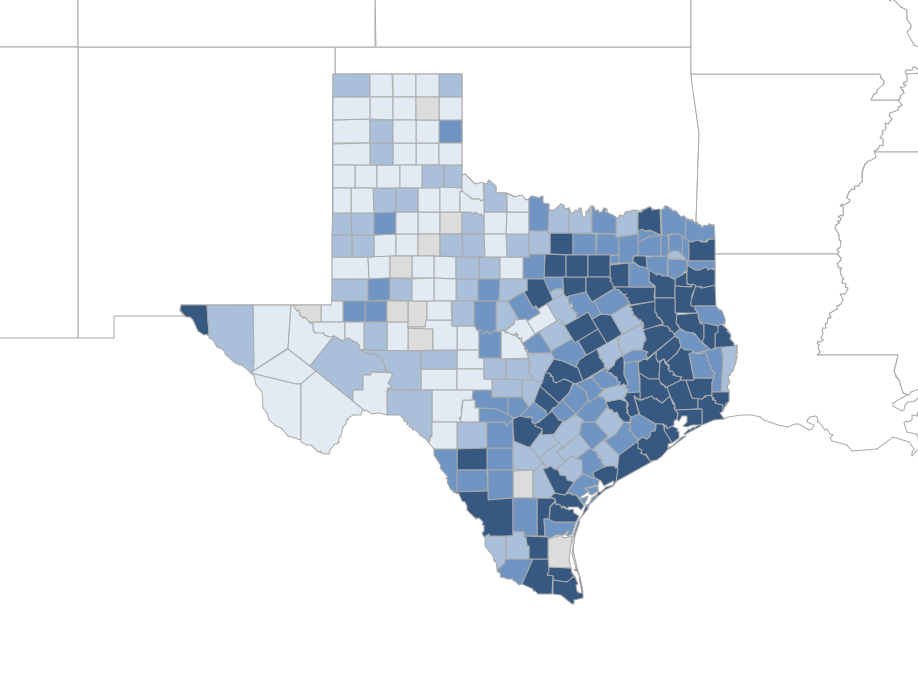

Supplement: Supplementary file 1 — Supplementary Material 1 [file 12872_2024_3749_MOESM1_ESM.docx]
